# Adenine Inhibits the Growth of Colon Cancer Cells via AMP-Activated Protein Kinase Mediated Autophagy

**DOI:** 10.1155/2019/9151070

**Published:** 2019-09-12

**Authors:** Hsin-Wu Lai, James Cheng-Chung Wei, Hung-Chang Hung, Chun-Che Lin

**Affiliations:** ^1^Institute of Medicine, Chung Shan Medical University, Taichung, Taiwan; ^2^Division of Gastroenterology, Department of Internal Medicine, Nantou Hospital, Ministry of Health and Welfare, Taipei, Taiwan; ^3^Division of Allergy, Immunology and Rheumatology, Department of Medicine, Chung Shan Medical University Hospital, Taichung, Taiwan; ^4^Graduate Institute of Integrated Medicine, China Medical University, Taichung, Taiwan; ^5^Department of Healthcare Administration, Central Taiwan University of Science and Technology, Taichung, Taiwan; ^6^Division of Gastroenterology, Department of Internal Medicine, Chung Shan Medical University Hospital, Taichung, Taiwan

## Abstract

**Background:**

Adenine is involved in a variety of cell biological processes and has been explored for pharmacological uses. Its therapeutic use for managing cancer is of great interest. In the present study, we investigated the anticancer effects of adenine and the underlying mechanism in colon cancer cells.

**Methods:**

Cell viability was measured using the MTT assay. Levels of phosphorylation and protein expression were determined using western blotting. qPCR was carried out to determine the changes in mRNA expression of genes of interest.

**Results:**

Adenine significantly inhibited the viability of colon cancer cells, HT29 and Caco-2 cells, in a dose-dependent manner. Adenine induced significant apoptosis in HT29 cells, whereas Caco-2 cells exhibited less apoptotic responses. The data showed that adenine activated AMP-activated protein kinase (AMPK) signaling contributing to autophagic cell death through mTOR in both colon cancer cell lines.

**Conclusions:**

Our findings suggest that adenine inhibits the growth of colon cancer cells. Anticancer activity of adenine in colon cancer cells is attributable to the activation of apoptotic signaling and in turn the AMPK/mTOR pathway. Adenine represents a natural compound with anticancer potency.

## 1. Introduction

Colorectal cancer (CRC) is one of the leading malignancies, which is expected to account for 2.2 million new cases and 1.1 million deaths worldwide in 2030 [1]. Approximately, 40% of patients with CRC present localized-stage disease at diagnosis, which is curable with surgical modality. 20% of CRC patients have metastasis at diagnosis. For patients with advanced CRC, treatment options include chemotherapy, radiation therapy, and biological therapy in combination with surgical modalities. Although these therapeutic regimens achieve somewhat satisfactory local disease control, effective regimens for metastatic CRC patients with liver metastases from colorectal cancer are limited.

Adenine is a purine derivative which is synthesized in the liver in humans. It also exists in foods such as brewer's yeast and vegetables. Adenine forms several biological compounds involved in a variety of cellular physiological processes such as adenosine triphosphate (ATP) in cellular respiration and deoxyribonucleic acid (DNA) in protein synthesis. Adenine also forms as a component of nicotinamide adenine dinucleotide (NAD) and flavin adenine dinucleotide (FAD), which are involved in metabolism. In addition to its physiological functions, adenine has also been demonstrated for its pharmacological properties. Recent studies have shown that adenine attenuates allergic responses [2, 3]. Adenine has been reported to exert anti-inflammatory activity in different experimental settings [4, 5]. Adenine improves the survival of rat Purkinje cells and enhances the storage of erythrocyte in whole blood [6–8]. Recently, adenine has been explored for its anticancer property in several types of cancer cell lines. Adenine has been suggested to induce cell cycle arrest in cancer cells, leading to cell death [9, 10]. However, the mechanism by which adenine inhibits the proliferation of cancer cells is sketchy. It is of interest to explore the inhibitory effect of adenine on the growth of colorectal cancer cells and to determine the underlying mechanism.

In this study, we investigated the effects of adenine against the proliferation of colon cancer cells. We explored the possible mechanisms underlying the anticancer activity of adenine in colorectal cancer cells. Inhibition of cell viability was assessed with focus on apoptosis transcriptionally and translationally. Involvement of AMP-activated protein kinase (AMPK) in anticancer property of adenine was also investigated.

## 2. Materials and Methods

### 2.1. Cell Culture

Human colon adenocarcinoma cell lines, HT29 (ATCC HTB-38) and Caco-2 (ATCC HTB37), were maintained and cultured in Dulbecco's Modified Eagle's Medium (DMEM, Gibco-BRL) supplemented with 10% fetal calf serum (FCS; Gibco-BRL) and 100 *μ*g/mL penicillin/streptomycin at 37°C. For treatment with adenine (Sigma-Aldrich, St. Louis, MO, USA), cells were initially seeded in 6-well culture plates at a density of 1 × 10^5^ cells/mL in a total volume of 2 mL and cultured overnight to reach approximately 80% confluence. Adenine treatments were carried out by incubating cells with adenine at designated concentrations (0, 0.1, 1, 5, and 10 mM) for 24 h or 48 h. The resulting cells were washed with phosphate-buffered saline (PBS) and collected for subsequent experiments.

### 2.2. Cytotoxicity Assay

The MTT assay was conducted to evaluate the cytotoxicity of adenine. In brief, cells were treated with adenine for 24 hours, and culture medium was subsequently aspirated, followed by incubation with MTT (0.5 mg/mL) at 37°C for 4 h. Removing the supernatant, formazan in culture was dissolved in isopropanol and analyzed spectrophotometrically at a wavelength of 563 nm. The number of viable cells was directly proportional to the concentration of formazan and was calculated in comparison with untreated cells.

### 2.3. Western Blotting

Cells were lysed using a lysis buffer containing 50 mM Tris-HCl, pH 7.5, 1% Nonidet P-40, 1 mM phenylmethylsulfonyl fluoride, and 1 mM NaF supplemented with protease inhibitor cocktail (Roche, Germany). The resulting cell lysate was centrifuged at 20,000 g for 15 min. Resulting supernatants were collected and assessed for protein concentration using the BCA method. 20 *μ*g of the crude protein was subjected to a 12.5% SDS-polyacrylamide gel and subsequently transferred onto a nitrocellulose membrane (Millipore, MA, USA). The resulting membranes were blocked with 5% (w/v) skimmed milk in PBS followed by incubation with primary antibodies (Cell Signaling Technology, MA, USA) at a ratio of 1 : 1000, which were against human cleaved caspase-3, cleaved caspase-8, poly (ADP-ribose) polymerase (PARP), phosphate-AMPK, AMPK, mammalian target of rapamycin (mTOR), phosphate-mTOR (p-mTOR), and glyceraldehyde 3-phosphate dehydrogenase (GAPDH), respectively. Blots were incubated with peroxidase-conjugated secondary antibodies, and antigen-antibody complexes were displayed using ECL chemiluminescence (Millipore, MA, USA).

### 2.4. Quantitative Real-Time PCR

Total RNA was extracted using TRIzol reagent (Ambion, CA) in accordance with the manufacture's instruction. RNA levels were measured using the Qubit® RNA Assay Kit (Invitrogen, CA). Reverse transcription was conducted in a 20 *μ*L reaction containing 200 ng of total RNA using cDNA reverse transcription kits (Applied Biosystems, CA), and quantification of genes of interest were evaluated using the ABI 7900HT system (Applied Biosystems) (Table 1).

### 2.5. Statistical Analysis

Student's *t*-test was performed to calculate the statistical significance between treatment groups and controls. Data were presented as mean ± SD of the three independent experiments using SigmaPlot 10 (Systat Software, San Jose, CA). *p* values less than 0.05 were considered statistically significant.

## 3. Results

### 3.1. Adenine-Inhibited Growth of Colorectal Cancer Cells

**Figure 1:**
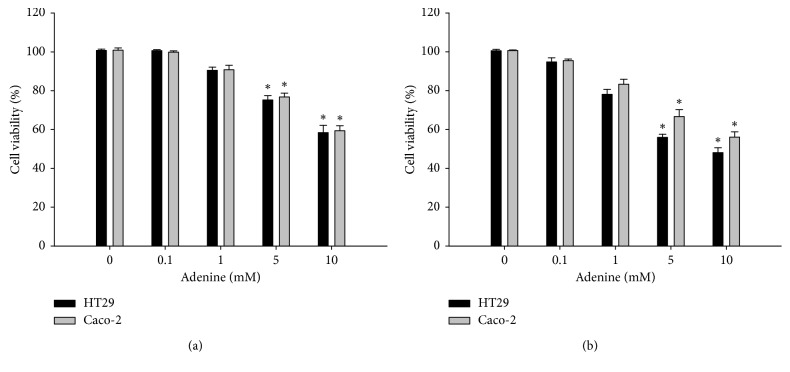
Adenine inhibited the growth of colorectal cancer cells. HT29 cells and Caco-2 cells were treated with adenine for 24 (a) and 48 (b) hours. Cell viability was evaluated using the MTT assay. Assay was conducted in triplicate, and the data are presented as mean ± SD. ^*∗*^*p* < 0.05 compared with 0 mM adenine.

Effects of adenine on the growth of colorectal cancer cells were determined in HT29 and Caco-2 cells using the MTT assay. HT29 and Caco-2 cells were treated with adenine at different concentrations and analyzed for viability. We found that adenine inhibited the growth of two colorectal cell lines in a dose-dependent manner, showing a significant decrease in viability to 58.4 ± 3.8% and 59.4 ± 2.6%, of the controls in presence of 10 mM of adenine for 24 hours, respectively (Figure 1). The prolonged treatment for 48 hours with adenine resulted in a greater inhibition of cell viability, which was 48.1 ± 2.5% and 56.1 ± 2.7% of the controls in presence of 10 mM of adenine, respectively. The 50% inhibitory concentration (IC50) was calculated for HT29 and Caco-2 cells, resulting as 2.838 mM and 22.198 mM, respectively.

### 3.2. Adenine-Induced Apoptosis in Colon Cancer Cells

**Figure 2:**
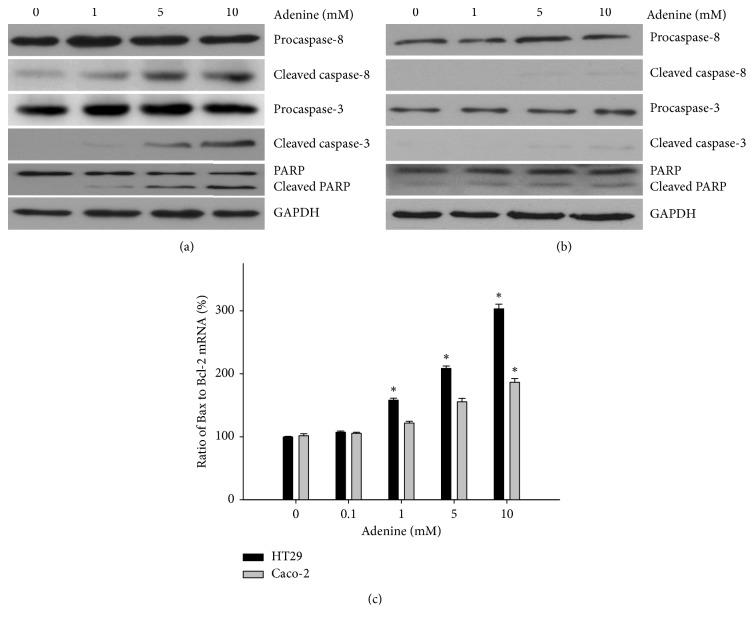
Adenine induces apoptosis in colon cancer cells. Colorectal cancer cells HT29 (a) and Caco-2 (b) were treated with serial concentrations of adenine for 24 hours. After treatment, total protein was extracted for western blotting. Primary antibodies against procaspase-3, caspase-3, procaspse-8, caspase-8, and PARP were used to measure the levels of apoptotic proteins. Total RNA extraction was performed, and cDNA was produced reverse-transcriptionally from mRNA for real-time PCR of Bax and Bcl-2 (c). Experiments were conducted in triplicate, and the data are presented as mean ± SD. ^*∗*^*p* < 0.05 compared with 0 mM adenine.

We next investigated the possible underlying mechanism of growth inhibition in both colorectal cancer cell lines in response to adenine treatment. We evaluated the induction of apoptosis based on the changes in levels of apoptotic biomarkers. As shown in Figure 2(a), a significantly increased level of cleaved caspase-3 was found in HT29 cells treated with 5 and 10 mM of adenine compared with that of controls. The results also showed that adenine treatment led to increased cleaved caspase-8 levels and significant cleavages of PARP in HT29 cells dose-dependently. Unexpectedly, Caco-2 cells were found to have relatively less induction of apoptosis in response to adenine treatment (Figure 2(b)). Given the activation of caspase cascades, we next investigated whether adenine treatment alters the expressions of apoptosis-related genes such as antiapoptotic protein Bcl-2 and the proapoptotic proteins. As shown in Figure 2(c), the ratio of Bax to Bcl-2 mRNA was remarkably increased in the cells treated with adenine at high concentrations in comparison with that of controls. Interestingly, HT29 cells were relatively more affected by adenine at high concentrations in comparison to Caco-2 cells (Figure 2(c)).

### 3.3. Adenine-Activated AMPK and -Regulated Autophagic Signaling in Colon Cancer Cells

**Figure 3:**
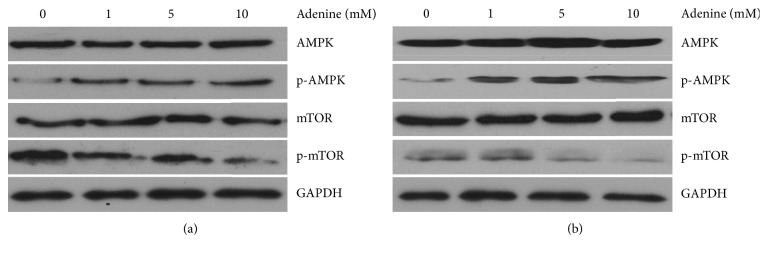
Adenine-induced AMPK activation and regulated AMPK signaling. Colon cancer cells HT29 (a) and Caco-2 (b) were treated with a serial concentration of adenine for 24 h and then were lyzed for the measurements of protein levels of p-AMPK, AMPK, p-mTOR, and mTOR using western blotting.

Adenine has been reported to upregulate AMPK signaling and in turn activate the biological activity [11, 12]. We hypothesized that adenine inhibits the growth of colon cancer cells through activating AMPK signaling. HT29 and Caco-2 cells were treated with adenine at serial concentrations, and degree of phosphorylation of AMPK and mTOR was analyzed. We found that treatment with adenine resulted in increased levels of AMPK phosphorylation in HT29 and Caco-2 cells in a dose-dependent manner (Figure 3). We also found that the increase in AMPK phosphorylation was associated with a decrease in the level of phosphate-mTOR.

**Figure 4:**
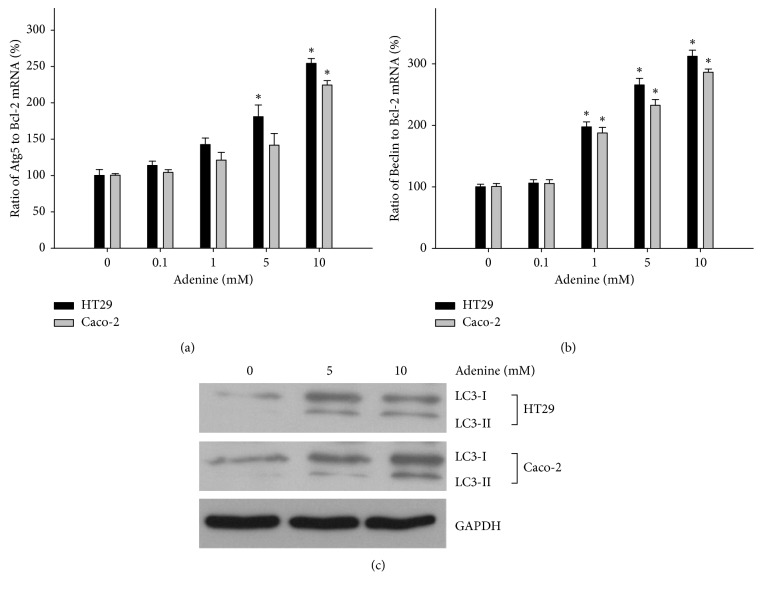
HT29 and Caco-2 cells were treated with serial concentrations of adenine for 24 hours. Total RNA extraction was performed, and cDNA was produced reverse-transcriptionally from mRNA for real-time PCR of Atg5 (a) and Beclin1 (b). Total proteins of adenine-treated cells were extracted for western blotting. Primary antibodies against LC3-1 and LC3-II were used to evaluate the degrees of autophagosome formation (c). Tests were conducted in triplicate, and the data are presented as mean ± SD. ^*∗*^*p* < 0.05 compared with 0 mM adenine.

It is known that autophagy is inhibited by mTOR. The role of autophagy in adenine-induced cell death in colon cancer cells was investigated. The results showed that exposure to adenine at concentrations of 5 mM and 10 mM led to an elevated ratio of Atg5 to Bcl-2 mRNA compared with that of controls in HT29 and Caco-2 cells, respectively (Figure 4(a)). The ratio of autophagy-related genes, Beclin1 and Bcl-2, in adenine-treated colon cancer cells was increased in a dose-dependent fashion (Figure 4(b)). The influence of adenine treatment on autophagosome was also determined as in the level of LC3-II using western blotting. Levels of LC3-II were significantly increased in association with increasing concentrations of adenine in HT29 and Caco-2 cells (Figure 4(c)).

### 3.4. Adenine Inhibited the Growth of Colon Cancer Cells via AMPK-Mediated Autophagy

**Figure 5:**
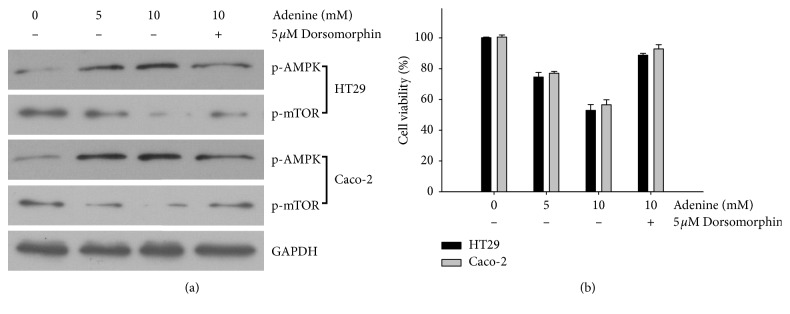
Adenine-induced autophagic inhibition on colon cancer cells via AMPK activation. (a) After pretreatment with dorsomorphin (5 *μ*M), colon cancer cells HT29 and Caco-2 were treated with adenine (10 mM) for 24 h . Levels of p-AMPK and p-mTOR were measured by western blotting. (b) Viabilities of cells receiving different treatments were evaluated using the MTT assay.

We found that AMPK activation was associated with induction of autophagy in presence of adenine; thus, effects of adenine-induced AMPK activation on the growth of colon cancer were investigated. The results showed that increased phosphorylation of AMPK in both colon cancer cell lines treated with adenine were restored in the presence of AMPK inhibitor, dorsomorphin, at a concentration of 5 *μ*M corresponding with increased mTOR phosphorylation (Figure 5(a)). Cotreatment with dorsomorphin significantly restored the viability of colon cancer cells treated with 10 mM adenine (Figure 5(b)).

## 4. Discussion

In the present study, we demonstrate that adenine inhibits the growth of human colon cancer cells via induction of apoptosis and autophagy. We found that adenine induced different degrees of apoptosis depending on the genotype of colon cancer cells. The inhibition of cell growth in colon cancer cells is associated with AMPK activation. Our results reveal that the inhibitory effect of adenine on proliferation of colon cancer cell is attributed to the activation of AMPK signaling.

Adenine, known as vitamin B4, has attracted much attention as a therapeutic agent based on its biochemical property. Its use has been explored as treatment for malignancies. Adenine has been demonstrated to induce apoptosis and cell cycle S-phase arrest in hepatocellular carcinoma cells and cervical cancer cells [9]. Chen et al. reported that adenine induced G2/M cell cycle arrest and autophagy in erythroleukemia K562 cells [10]. It is suggested that adenine-induced inhibition of cancer cell growth varies greatly depending on the type of cancer cells. Apoptosis plays a critical role in cancer development. Abnormal apoptosis is considered to contribute to the pathogenesis of colorectal cancer and resistance to treatments in particular. In this study, we found that HT29 cells are relatively more sensitive to adenine treatment than Caco-2 cells as a result of different degree of apoptotic responses. Our findings showed that adenine treatment led to increased mRNA ratio of Bax to Bcl2 and elevated levels of cleaved caspase-3 and caspase-8, suggesting that adenine could affect the expression of Bcl2 proteins and activate apoptotic pathway in HT29 cells. Our data indicate that adenine induced significant apoptosis in colorectal adenocarcinoma HT29 cells, a p53 mutant, whereas Caco-2 cells, a p53-null cell line, exhibited less apoptosis. This finding is in agreement with the previous study showing adenine fails to induce apoptosis in leukemia K562 cells which are p53-negative [10]. It is suggested that p53 status may play a role in responsiveness to adenine treatment in the aspect of apoptosis. In addition, treatment with adenine for 48 hours was shown to have a relatively high inhibitory rate in HT29 compared with Caco-2 cells, suggesting that the other cell death processes may contribute to the adenine-induced inhibition in colon cancer cells such as autophagy. Further studies are necessary to elucidate the involvement of p53 in adenine-associated inhibition of colon cancer cells.

AMP-activated protein kinase is known for its role in regulation of the protein and lipid metabolism as a conserved energy sensor [13]. Recently, AMPK signaling has been considered as a target process for cancer prevention and therapy [14]. One of the growth signaling pathways regulated by AMPK is the mammalian target of rapamycin (mTOR) pathway, which controls a variety of biological processes involved in cell survival, migration, and metabolism. Activation of AMPK has been shown to suppress the mTOR signaling leading to, in part, the activation of autophagy. Several studies have reported that activation of AMPK induces autophagic cell death in various cancer cells via suppression of mTOR [15–19]. Exogenous adenine increases the expression and translocation of glucose transporter 4, enhances the cellular glucose uptake, and elevates the intracellular ATP level [11]. Adenine has been demonstrated to induce AMPK activation in different types of cells [12, 20–22]. A recent study has shown that adenine inhibited the growth of leukemia K562 cells via AMPK activation. In our study, we demonstrated that adenine significantly induced AMPK activation and inhibited the phosphorylation of downstream mTOR in both colon cancer cell lines. In addition, we showed an increased level of LC3-II with corresponding elevated expressions of Atg5 and Beclin in HT29 and Caco-2 cells after exposure to adenine. The findings suggest that adenine triggers autophagic cell death in colon cancer cells in present setting. Adenine-inhibited cell proliferation in both colon cancer cell lines was restored with increased levels of mTOR phosphorylation in the presence of AMPK inhibitor dorsomorphin. These findings are in consistent with the previous study showing adenine-induced autophagy via AMPK signaling corresponding with changes in the mTOR level in leukemia [10]. Autophagy has been shown to have dual and contradictory roles in carcinogenesis. In addition to cell death, autophagy is shown to provide a backup energy source for the survival and expansion of tumor cells, particularly cancers at advanced stages [23–26]. In the present experimental setting, it is indicated that adenine activates AMPK subsequently contributing to autophagic cell death in colon cancer cells. Nevertheless, treatment with dorsomorphin incompletely reversed the inhibited cell growth, suggesting that adenine may suppress colon cancer cells via AMPK-independent pathways.

In conclusion, we show evidence that adenine significantly inhibits the proliferation of colorectal cancer cells through synergistically in part apoptosis and autophagy. The adenine-induced autophagic cell death is AMPK-dependent in colon cancer cells, whereas apoptosis is attributed to p53 status. By manipulating both arms of apoptotic and autophagic signaling, adenine represents a promisingly effective therapeutic agent against human colorectal cancers. Further studies are required to elucidate the role of p53 in adenine-induced apoptosis in colon cancer cells.

## Figures and Tables

**Table 1:** 

Gene	Forward primer (5'–3')	Reverse primer (3'–5')
Bax	AGTGGAGCTGCAGAGGATGA	ATGGCCTTGAGCACCAGTTT
Bcl-2	ATAACTGGAGAGTGCTGAAGA	ATGTTGTATTTTTTAAGTACAGC
Atg5	ATGGACAGTTGCACACACTA	TCTTCAGGATCAATAGCAGAA
Beclin1	CTCACAGCTCCATTACTTACCA	CAATAAATGGCTCCTCTCCTGA
GAPDH	AATGGAAATCCCATCACCATCT	CAGCATCGCCCCACTTG

## Data Availability

The data used to support the findings of this study are included within the article.
